# Meet key Digital Health thought leaders

**DOI:** 10.1093/ehjdh/ztab070

**Published:** 2021-09-14

**Authors:** Bruining Nico

**Affiliations:** Erasmus MC, Thoraxcenter, Department of Cardiology, Dr. Molewaterplein 40, 3015GD Rotterdam, The Netherlands

**Martin Cowie, MD, talks to CardioPulse Digital about his fascination and commitment to Digital Health.**

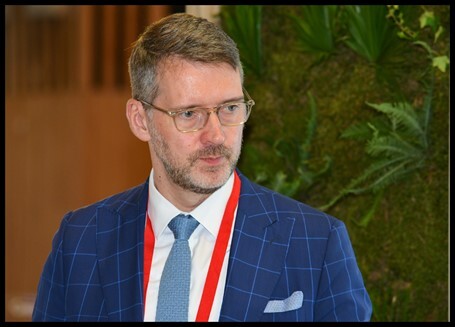

Martin Cowie is Professor of Cardiology at the Royal Brompton Hospital in London (UK), where he has been leading the heart failure service for the past 20 years. During his medical training, he became fascinated by heart failure, despite diagnosis, treatment, and monitoring at that time being in their infancy. Over the past 15 years, he has been investigating the value of remote monitoring of heart failure, in large randomized studies. From 2016 to 2020, he was Non-Executive Director of the National Institute for Health and Care Excellence (NICE) in England, where he learned first-hand the challenges and opportunities of thoroughly assessing the clinical value and cost-effectiveness of new diagnostics, medicines, and devices, and ways of working. He speaks to CardioPulse Digital as the chair of the European Society of Cardiology—Digital Health Committee.

## You chair the Digital Heath Committee of the ESC. Can you tell us more about the committee’s achievements?

Since the ESC published its Position Paper on Digital Health in 2016,^1^ I have helped co-ordinate increased activity in the digital space. This culminated in the inauguration of a Digital Health Committee in 2018. Digital Health is a very wide topic, and in a sense, most health and healthcare is already digital. We focus on the use of information and communication technology in the support of health and healthcare. This includes electronic medical records and e-prescribing, remote monitoring and consultation, mobile health approaches (basically anything that can use a smartphone or similar device), and links in with Big Data initiatives. The Committee has a broad representation from the ESC and advisors from the patient community and life-science industry. The key deliverables include an Annual Digital Health Summit (next on 22–24 October 2021: please register on https://www.escardio.org/Congresses-&-Events/ESC-Digital-Summit/Registration); good representation of digital health topics at the Annual main ESC congress; the launch of our journal ‘EHJ-Digital Health’ in 2020; and helping the ESC work through the issues related to mobile health (including Apps) and the ethics of Artificial Intelligence.

## What fascinates you about Digital Health?

I have always been interested in using technical innovations to improve cardiovascular care. Sadly, most people seem to divide into one of two camps: enthusiasts/evangelists or sceptics. I strongly believe that we need to embrace innovation if proven to be an improvement: but just because it is new and “shiny” does not make it valuable. Ensuring the clinical voice is heard, along with the patient perspective, is vital to a mature conversation about the co-design and evaluation of new digital approaches. But to a large extent, this is the ‘new normal’: I dislike the word “disruption” as it often causes people to react defensively, but many digital approaches and tools can help support better care, better experience, and better outcomes. But not all.

## What makes Digital Health successful?

In my opinion, most aspects of modern life are easier and more convenient when a digital approach is part of a set of tools. Booking flights, managing your finances, communicating with friends and colleagues is all largely digital, but healthcare is slow to embrace this. Of course, the risk of harm must be avoided or at least limited. However, there is also conservatism among healthcare professionals and regulators, and finances are often an additional barrier. Success comes when there is co-design with those who will adopt the technology. A supportive training environment, professional, and patient support, and fees to facilitate adoption are additional requirements. In cardiovascular disease, this often requires robust evidence—not necessarily always from large randomized trials—combined with a sense of ownership at the local level. Achieving these things is challenging, but tech developers and investors are becoming more realistic about what it takes for further Digital Health adoption!

## What disruptive Digital Health innovations do you see on the horizon?

I would move the focus from the horizon to closer to now: already artificial intelligence (AI) and its underpinning machine learning approaches have transformed image interpretation and diagnostics, and will increasingly support better and more personalized (and broadly based) decision-making and prognostication. Remote monitoring and consultation have undergone a ‘techcelleration’ with the COVID-19 pandemic and we are unlikely to return to anything other than a hybrid approach of face-to-face contact complemented with more remote support. Also empowering those living with chronic conditions using novel technology to support their own management is rapidly improving. It blurs the previously clear lines between ‘healthcare’ and the ‘consumer’.

## Is there anything else you would like to share as a Digital Health Ambassador?

I would like to take the opportunity to encourage all readers of this journal—from whichever walk of life—to support the ESC and its Digital Health initiatives. Let’s make the future together!

**Conflict of interest:** The author declares no conflict of interests.
